# Summary of the best evidence for physical therapy in patients with post-stroke shoulder hand syndrome

**DOI:** 10.3389/fneur.2026.1779579

**Published:** 2026-04-02

**Authors:** Ganlu Tian, Jing Li, Fei Yao, Xin Wang

**Affiliations:** Department of Nursing, Beijing Tiantan Hospital, Capital Medical University, Beijing, China

**Keywords:** evidence summary, evidence-based nursing, physical therapy, shoulder-hand syndrome, stroke

## Abstract

**Background:**

Shoulder-hand syndrome (SHS) is a common complication after stroke, characterized by shoulder pain, hand swelling, and limited mobility, which severely impacts patient rehabilitation and quality of life. Physical therapy is central to its comprehensive management, yet clinical intervention approaches vary and lack standardization.

**Objective:**

To systematically retrieve, evaluate, and integrate the best evidence on physical therapy and nursing for post-stroke SHS, providing a basis for clinical practitioners to develop standardized management protocols.

**Methods:**

Following evidence summary methodology and the “6S” pyramid model, we systematically searched domestic and international databases, guideline repositories, and professional association websites. The search period covered from database inception to 1 December 2025. Two researchers independently assessed the quality of the literature using the AGREE II instrument for guidelines and the relevant Joanna Briggs Institute (JBI) critical appraisal tools for other study types. Evidence was subsequently extracted and summarized.

**Results:**

The initial search identified 734 records. After screening, 22 articles were included, comprising 4 guidelines, 1 clinical decision, 14 systematic reviews, 1 evidence summary, and 2 expert consensus. Ultimately, 18 best practice recommendations were synthesized across 6 themes: early prevention and protection, comprehensive assessment, symptom management, transfers and mobility, posture management, and health education and follow-up.

**Conclusion:**

This study summarizes the best available evidence for the physical therapy and nursing management of post-stroke SHS, forming a comprehensive strategy covering prevention, assessment, intervention, and health education. It provides an evidence-based foundation for standardizing clinical practice and enhancing patient recovery.

**Systematic review registration:**

This study was based on the evidence summary reporting specifications of the Fudan University Center for the Evidence-based Nursing, the register name is “Summary of the best evidence for physical therapy in patients with post-stroke shoulder hand syndrome”, the registration number is “ES20258280”.

## 1 Introduction

Stroke is one of the leading causes of disability worldwide. More than 30 to 50% of stroke patients will experience varying degrees of upper limb dysfunction, with motor and sensory impairments being particularly prominent (1, 2). Shoulder-hand syndrome (SHS), also known as Complex Regional Pain Syndrome type I (CRPS-I) or reflex sympathetic dystrophy, is a common complication following stroke (3). Its typical clinical manifestations include pain in the affected shoulder, swelling of the wrist or hand, elevated skin temperature, and restricted range of motion (4). Without timely and effective intervention, it may progress to muscle atrophy in the hand and shoulder, joint contractures, and even permanent disability, significantly compromising the patient’s quality of life.

The pathogenesis of SHS has not been fully elucidated. It is generally believed to involve three main mechanisms:

(i) *Neurological mechanisms*: Central nervous system injury following stroke can lead to abnormal sympathetic excitation, resulting in impaired peripheral vasoconstriction, reduced local tissue perfusion, and dysregulated pain signaling, ultimately contributing to neuropathic pain and sensory abnormalities.
(ii) *Vascular and inflammatory mechanisms*: Limb immobility after stroke causes impaired venous return and increased release of inflammatory mediators, leading to microcirculatory dysfunction, localized tissue edema, and hyperalgesia.
(iii) *Biomechanical factors*: Glenohumeral subluxation, abnormal movement patterns, and muscle spasm may cause recurrent microtrauma to joints and periarticular soft tissues, establishing a vicious cycle of “pain–immobility–atrophy” (5, 6). Given its complex and often progressive clinical presentation, early recognition, prevention, and evidence-based intervention are essential.

Physical therapy serves as the cornerstone of comprehensive management for shoulder-hand syndrome (SHS). It employs a range of non-invasive, function-oriented interventions aimed at alleviating pain, reducing edema, improving joint range of motion, enhancing muscle strength, and breaking the vicious cycle of symptoms—ultimately leading to improved limb function. However, in clinical practice, physical therapy modalities are highly diverse, including rehabilitation exercises (7), acupuncture (8), transcutaneous electrical nerve stimulation (TENS) (9), mirror therapy (10), extracorporeal shockwave therapy (ESWT) (11), among others. The strength of evidence supporting their efficacy varies, and there is a lack of standardized clinical guidelines. As a result, healthcare providers often face challenges in selecting the optimal intervention strategy.

## 2 Materials and methods

### 2.1 Criteria for summarizing evidence

The evidence synthesis followed the evidence synthesis report standards of the Evidence-Based Nursing Center at Fudan University, including problem formulation, evidence retrieval, literature screening, literature quality evaluation, evidence synthesis, and grading (12)(Registration No. ES20258280). Literature search, screening, quality assessment, and evidence synthesis were performed independently by two researchers. Any discrepancies were resolved through consensus or by consultation with the corresponding author.

### 2.2 Establishment of the problem

Based on the PIPOST model, construct evidence-based questions:

P (Population): The target population for evidence implementation is adult stroke patients (≥18 years) with concurrent shoulder-hand syndrome.
I (Intervention): Interventions of interest include physical therapy modalities such as acupuncture, transcutaneous electrical nerve stimulation (TENS), mirror therapy, and extracorporeal shock wave therapy (ESWT).
P (Professionals): Healthcare professionals involved in evidence application, particularly clinicians, nurses, and allied health practitioners.
O (Outcomes): Key outcomes include pain intensity scores, quality of life assessments, and functional performance in activities of daily living (ADL).
S (Setting): Applicable settings include hospitals, medical rehabilitation institutions, and community-based healthcare environments.
T (Type of evidence): Evidence types include clinical decision support resources, clinical practice guidelines, systematic reviews, expert consensus documents, and evidence summaries.

### 2.3 Criteria for considering studies

Inclusion criteria were as follows: (i) studies involving patients with post-stroke shoulder-hand syndrome; (ii) interventions focusing on physical therapy modalities; (iii) literature types were clinical guidelines, Best Practice, Evidence Summaries, expert consensus, systematic review; (iv) language limited to Chinese or English.

Exclusion criteria comprised: (i) articles with incomplete data or unavailable full text; (ii) translated or interpreted versions of guidelines; (iii) studies with critically low methodological quality.

### 2.4 Search strategy

This study conducted a comprehensive literature search following the 6S Pyramid model in a top-down manner (13). Evidence-based resources searched included: UpToDate, the National Institute for Health and Care Excellence (NICE) guidelines, the Guidelines International Network (GIN), the Scottish Intercollegiate Guidelines Network (SIGN), the Registered Nurses’ Association of Ontario (RNAO), the Cochrane Library, Web of Science, PubMed, Embase, CINAHL, DynaMed, and Scopus. Search terms encompassed “Stroke/Cerebral Stroke/Cerebrovascular Apoplexy,” “Reflex Sympathetic Dystrophy/Reflex Sympathetic Dystrophy Syndrome/Cervical Sympathetic Dystrophy/Shoulder Hand Syndrome,” and “Physical Therapy Modalities/Physical Therapy Modality/Neurophysiotherapy/Physical Therapy/Rehabilitation.” A combination of subject headings and free-text terms was employed, with the search period covering records from the establishment of the database until 1 December 2025. We used the search strategy described in the Supplementary Table 1.

### 2.5 Quality evaluation of the literature

(1) *Clinical guidelines*: The Appraisal of Guidelines for Research and Evaluation II (AGREE II) instrument was used for guideline evaluation (14). It comprises 23 items, each rated on a 7-point scale (1 = strongly disagree to 7 = strongly agree). Domain scores were calculated as the sum of individual item scores within each domain and then standardized using the formula: [(obtained score – minimum possible score)/(maximum possible score – minimum possible score)] × 100% (15). Guidelines were categorized as: Grade A (all six domains ≥60% – recommended for use), Grade B (at least three domains ≥60%, with some domains potentially <60% – recommended after modifications), or Grade C (three or more domains <30% – not recommended).

(2) *Clinical decisions and evidence summaries*: Critical For Summaries of Evidence checklist (CASE) was used for evaluation (16). This tool generally consists of 14 items, with response options such as “yes,” “no,” or “partially” for each item.

(3) *Systematic reviews and meta-analyses*: This study employed the AMSTAR 2 systematic evaluation tool (17). The tool comprises 16 items, of which 7 are key items (items 2/4/7/9/11/13/15). Literature with no or only one non-key item non-compliance was considered high-quality; literature with one or more non-key item non-compliance was considered medium-quality; literature with only one key item non-compliance was considered low-quality; literature with more than one key item non-compliance was considered very low-quality.

(4) *Expert consensus articles*: The JBI Critical Appraisal Checklist for Text and Opinion Papers was used (18). It includes six items, each appraised as “yes,” “no,” “unclear,” or “not applicable.” Final inclusion, exclusion, or the need for further information was determined through panel discussion.

### 2.6 Evidence extraction, integration and evaluation

Two researchers extracted, categorized, and synthesized the evidence from the final selected literature. Using the Australian JBI Evidence-Based Healthcare Center’s Evidence Classification (2014 edition), they classified the evidence into five levels, with higher evidence grades indicating more rigorous study designs (1a being the highest and 5c the lowest). Additionally, the research team applied the JBI FAME framework—which evaluates the feasibility, appropriateness, meaningfulness, and effectiveness of evidence—to rate each piece of evidence and assign a recommendation grade of either Grade A (strong recommendation) or Grade B (weak recommendation), thereby ensuring a systematic and consistent evaluation process.

## 3 Results

### 3.1 Search results

**Figure 1:**
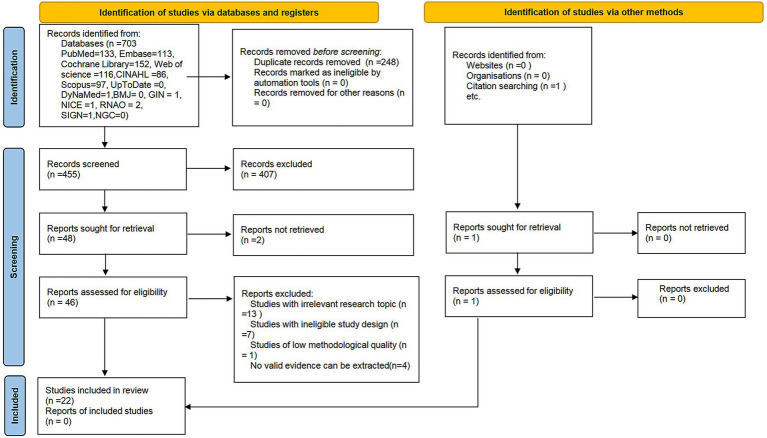
Article selection process: PRISMA flow diagram.

A total of 734 records were identified through the initial search. After a tiered screening process, 22 studies met the eligibility criteria for final inclusion. The study selection process is detailed in Figure 1.

### 3.2 General characteristics of included literature

A total of 22 articles were included in this study, including 4 clinical guidelines; 1 clinical decision; 14 systematic reviews; 1 evidence summaries and 2 expert consensus. Specific information is shown in Table 1.

**Table 1.** General characteristics of literature.

**Inclusion in the literature**	**Year of publication (year)**	**Source of the article**	**Type of literature**	**The literature theme**
National Institute for Health and Care Excellence (19)	2024	NICE	Guidelines	Stroke rehabilitation in adults
Intercollegiate Stroke Working Party (20)	2023	SIGN	Guidelines	National clinical guideline for stroke
Guidelines International Network (21)	2025	GIN	Guidelines	Australian and New Zealand Living Clinical Guidelines for Stroke Management
Salbach et al. (22)	2019	Pubmed	Guidelines	Canadian Stroke Best Practice Recommendations
Fan et al. (23)	2025	Dynamed	Clinical practice	Long-Term Management of Stroke
Saikaley et al. (24)	2023	EBRSR	Evidence summary	Hemiplegic Shoulder Pain and Complex Regional Pain Syndrome
Liu (8)	2019	Pubmed	Systematic review	Acupuncture for Post-stroke Shoulder-Hand Syndrome: A Systematic Review and Meta-Analysis
Peng (25)	2017	Pubmed	Systematic review	Traditional manual acupuncture combined with rehabilitation therapy for shoulder hand syndrome after stroke within the Chinese healthcare system: a systematic review and meta-analysis
Lei (26)	2022	Pubmed	Systematic review	Acupuncture for shoulder-hand syndrome after stroke: An overview of systematic reviews
Shi (27)	2025	Pubmed	Systematic review	Acupuncture versus rehabilitation for post-stroke shoulder-hand syndrome: a systematic review and meta-analysis of randomized controlled trials
Huang (28)	2023	Pubmed	Systematic review	Effectiveness of acupuncture for pain relief in shoulder-hand syndrome after stroke: a systematic evaluation and Bayesian network meta-analysis
Wei (29)	2019	Pubmed	Systematic review	Electroacupuncture for Reflex Sympathetic Dystrophy after Stroke: A Meta-Analysis
Wang (30)	2021	CINAHL	Systematic review	Network Meta-analysis of 4 acupuncture therapies for shoulder hand syndrome after stroke
Yu (31)	2021	CINAHL	Systematic review	Effect of moxibustion on rehabilitation of patients with shoulder-hand syndrome after stroke: a Meta-analysis
Shafiee (32)	2018	Pubmed	Systematic review	The Effectiveness of Rehabilitation Interventions on Pain and Disability for Complex Regional Pain Syndrome: A Systematic Review and Meta-analysis
Kanika (33)	2023	Pubmed	Systematic review	Effectiveness of the physiotherapy interventions on complex regional pain syndrome in patients with stroke: A systematic review and meta-analysis
Feng (34)	2022	Pubmed	Systematic review	EMG biofeedback combined with rehabilitation training may be the best physical therapy for improving upper limb motor function and relieving pain in patients with the post-stroke shoulder-hand syndrome: A Bayesian network meta-analysis
Gao (35)	2025	Pubmed	Systematic review	Effectiveness of acupuncture and moxibustion combined with rehabilitation training for post-stroke shoulder-hand syndrome: a systematic review and meta-analysis.
Meng (36)	2025	Pubmed	Systematic review	Efficacy and safety of moxibustion treatment for upper extremity pain disorder and motor impairment in patients with stage I post-stroke shoulder-hand syndrome: a systematic review and meta-analysis of randomized controlled trials
Wang (37)	2025	Pubmed	Systematic review	Efficacy and safety of acupuncture or moxibustion combined with rehabilitation therapy for post-stroke shoulder-hand syndrome: a systematic review and meta-analysis of randomized controlled trials
Maso (41)	2025	Pubmed	Expert consensus	A physiotherapy protocol* for stroke patients in acute hospital settings: expert consensus from the Brazilian early stroke rehabilitation task force
Castelnuovo (43)	2016	Pubmed	Expert consensus	Psychological Treatments and Psychotherapies in the Neurorehabilitation of Pain: Evidences and Recommendations from the Italian Consensus Conference on Pain in Neurorehabilitation

### 3.3 Literature quality evaluation results

#### 3.3.1 Guidelines quality evaluation results

This study included four guidelines (19–22) that scored ≥60% in each field and were rated as grade A. Consequently, these guidelines are considered high quality and are included in the summary. The results of the guideline evaluation are shown in Table 2.

**Table 2:** Guidelines Quality Evaluation Results.

Guideline	Standardized scores in various domains (%)								
	Scope and purpose	Stakeholder involvement	Rigor of development	Clarity of presentation	Applicability	Editorial independence	≥ 60%	≥ 30%	Quality evaluation
National Institute for Health and Care Excellence (19)	83.33%	88.89%	83.33%	83.33%	95.83%	83.33%	6	6	A
London: Intercollegiate Stroke Working Party (20)	86.11%	91.67%	86.46%	88.89%	95.83%	91.67%	6	6	A
Guidelines International Network (21)	86.11%	91.67%	89.58%	86.11%	97.92%	91.67%	6	6	A
Salbach et al. (22)	83.33%	91.67%	87.50%	77.78%	97.92%	87.50%	6	6	A

#### 3.3.2 Clinical decision and evidence synthesis quality evaluation results

This study included one clinical decision (23) and one evidence summary (24). Regarding the clinical decision evaluation, no relevant literature search information was found, so the answer to ‘4. Are the search methods transparent and comprehensive?’ was ‘No’, while all other items were answered ‘Yes’. For the evidence summary evaluation, all items were answered ‘Yes’. Given these results, the two articles were considered high quality and included in the study. Detailed evaluation results are presented in Supplementary Table 2.

#### 3.3.3 Systematic reviews quality evaluation results

This study included 14 systematic reviews published over the past 10 years (8, 25–37), all concerning physical therapy for post-stroke shoulder-hand syndrome. These encompassed various physical therapies such as acupuncture (including conventional acupuncture, electroacupuncture, fire needle therapy, and warm needle therapy), moxibustion, and rehabilitation training. Methodological quality was assessed using the AMSTAR 2 tool. As shown in Supplementary Table 3, eight studies (8, 25, 27, 28, 34–37) were rated as high quality, one study (26) as medium quality, and five studies (29–33) as low quality. Despite some methodological limitations—such as lack of a prior protocol, inadequate handling of heterogeneity, or failure to assess publication bias—all 15 studies met the minimum criteria for inclusion in this evidence synthesis.

#### 3.3.4 Expert consensus quality evaluation results

The study included only two expert consensus statements (26–25), which was rated as ‘Yes’ for all quality assessment criteria, thus deemed high-quality and eligible for inclusion.

### 3.4 Evidence integration results

The study extracted and summarized 18 pieces of evidence, which we divided into 6 themes, as shown in Table 3.

**Table 3:** Summary of best evidence for physical therapy for post-stroke shoulder hand syndrome.

Evidence topic	Evidence content	Evidence level	Recommendation grade
Early-phase shoulder recovery – prevention	1. In the early recovery phase, joint protection strategies are recommended to prevent or minimize local pain and injury (22): (i) Maintain correct arm positioning and provide support at rest; (ii) avoid pulling the affected arm during functional activities; (iii) provide protective support during wheelchair use, e.g., half-tray, arm trough, or protective pillow; (iv) sling use is not recommended unless upper limb muscle weakness is present; (v) active, active-assisted, or passive movements are recommended to prevent shoulder-hand syndrome.	Level 5b	A
Assessment	2. It is recommended to use validated tools such as the Visual Analog Scale (VAS), joint swelling score, and Fugl-Meyer Assessment before, during, and after treatment to regularly assess and record pain, joint swelling, and upper limb function in patients with shoulder-hand syndrome (8, 20–30, 41, 43).	Level 1a	A
	3. Prior to intervention, assess the patient for musculoskeletal issues, subluxation, and spasticity (24).	Level 5b	A
	4. Assess muscle tone, active movement, soft tissue length changes, shoulder girdle joint alignment, trunk posture, pain level, orthopedic shoulder changes, and the impact of pain on physical and emotional well-being (22).	Level 5b	B
Symptom management	5. It is recommended to adopt a phased and targeted combination therapy approach. By tailoring a comprehensive rehabilitation plan based on the severity of the condition and the main symptoms, the clinical efficacy can be enhanced (19).	Level 1a	A
	6. To alleviate shoulder pain and improve limb function, multimodal interventions may be employed, including exercise interventions, neuromuscular electrical stimulation (33), shoulder girdle stabilization, various forms of acupuncture (20, 28, 31, 33, 35, 37), moxibustion (37, 38), mirror therapy (36–35), and psychological care (8, 21, 24, 25, 27, 32, 43), electroacupuncture therapy (32, 34), administered 5–7 times weekly for 30 min each session, for 4 weeks (29); mirror therapy 30–60 min, 5 times a week for 4 weeks (33).	Level 1a	A
	7. For patients with shoulder subluxation or arm weakness within 6 months after stroke, a personalized neuromuscular electrical stimulation program should be developed based on specific symptoms and tolerance, provided there are no contraindications (20, 22).	Level 1a	A
	8. For patients with persistent local pain post-stroke, consider orthotic devices and spasticity management, with specialist consultation or referral if necessary (20).	Level 5b	A
	9. For the cases with limited range of motion, gentle stretching and mobilization techniques should be used to gradually increase the external rotation and abduction movements. While gradually expanding the active range of motion, the alignment of the shoulder joint should be corrected and the weak muscle groups should be strengthened. Local bandaging of the affected shoulder may help relieve the pain (22).	Level 5b	A
	10. For hand edema, consider active, active-assisted, or passive range-of-motion exercises, elevation of the upper limb on pillows (41), elevation of the affected arm at rest when possible, massage, and gentle Grade I-II accessory mobilization to the hand and fingers (22).	Level 5b	A
Transfers and mobility	11. Be careful to position and support the affected limb during movements such as assisted transfer (22).	Level 5b	A
	12. During the transfer, do not pull the patient’s upper limb, and pay attention to the range of motion to avoid injury (41).	Level 5b	A
Posture management	13. For patients with arm weakness, in the absence of scapular upward rotation and humeral lateral rotation, the passive range of motion of the shoulder joint for arm flexion and abduction should not exceed 90 degrees (19, 20, 22).	Level 5b	A
	14. Pay attention to the position of the body, adjust the position of the upper limb to get full support, and ensure that the upper limb does not hang or hangs partially (20, 22, 41).	Level 5b	A
	15. Avoid using head-mounted arm supports, shoulder support belts and pulley devices (20).	Level 5b	B
Health education	16. Provide information to stroke patients, their families, and caregivers on how to prevent shoulder pain or trauma health education (19)	Level 5b	A
	17. Educate patients and their families on the correct methods for protecting, positioning, and handling the affected limb (22).	Level 5b	A
	18. If electrical stimulation therapy is involved, patients, family members, and related nursing staff should receive training on the safe use of electrical stimulation devices (20).	Level 5b	A

## 4 Discussion

This study systematically synthesized 18 evidence items for the prevention, assessment, and management of SHS following stroke, providing a foundation for developing standardized physical therapy and nursing protocols. The evidence underscores that SHS management should constitute a multidimensional, dynamic, and continuous process—spanning from early prevention to long-term symptom control.

### 4.1 The management of post-stroke shoulder-hand syndrome should start with early prevention

Early implementation of protection strategies is crucial for preventing SHS after stroke (38). Based on Evidence Item 1, key protective measures include maintaining proper positioning, avoiding shoulder traction during activities, and using assistive devices. The core objective is to prevent periarticular soft tissue damage and secondary pain by eliminating mechanical stress, thereby interrupting the initial phase of SHS. Notably, this evidence item explicitly advises against the routine use of slings in patients whose only impairment is upper limb weakness (22). This reflects the rehabilitation principle of “active prevention” over “passive restriction,” thereby avoiding joint contractures and muscle disuse associated with prolonged immobilization.

### 4.2 The assessment of post-stroke shoulder-hand syndrome should be conducted from multiple perspectives

Scientific intervention must be grounded in precise assessment. Evidence Items 2–4 form a progressive assessment framework. Evidence Item 2 emphasizes the use of standardized tools such as the Visual Analog Scale and Fugl-Meyer Assessment (39) for dynamic monitoring, enabling objective quantification of pain, edema, and functional status to reliably evaluate treatment efficacy (20, 30). Evidence Item 3 focuses on pre-intervention safety screening to identify key issues such as subluxation and spasticity that may influence treatment selection and safety (20). Evidence Item 4 further deepens the assessment by incorporating factors ranging from muscle tone and joint alignment to emotional impact (22), laying a solid foundation for developing comprehensive care plans based on the biopsychosocial model.

### 4.3 The intervention for shoulder-hand syndrome after a stroke should be targeted

The symptom management in SHS is a dynamic and systematic process. Evidence item 5 states that the treatment for SHS after stroke should follow the core principle of “phased, targeted combined therapy” (20) Item 6 indicates that multiple physical therapies such as exercise intervention, acupuncture, neuromuscular electrical stimulation, and mirror therapy may be used in patients with post-stroke shoulder-hand syndrome. It also specifies treatment parameters for electroacupuncture and mirror therapy, providing key details for standardizing clinical practice (33). Evidence Items 7–10 list targeted interventions for various clinical symptoms of patients with post-stroke shoulder-hand syndrome. It is worth noting that the included evidence covers two types: conventional physical therapy and traditional Chinese medical techniques (such as acupuncture and moxibustion). Although our comprehensive analysis suggests that both these methods may bring clinical benefits to the management of SHS, the application effects of traditional Chinese medical techniques like acupuncture often depend on specific acupoints and techniques, and may be difficult to replicate in non-Chinese environments. Therefore, clinical practice should be individualized according to local resources and patient preferences. Evidence item 7 addresses specific issues such as joint subluxation and arm weakness. It suggests that an individualized neuromuscular electrical stimulation program can be adopted, which has certain positive effects in restoring neuromuscular control, stabilizing the joints, and promoting functional recovery (20, 22). For refractory shoulder pain that does not respond to conventional treatment, evidence Item 8 recommends consideration of anti-spasmodic treatment. For example, an orthosis can be used to maintain a spastic limb in a functional or anti-spastic position. This applies a continuous, stable, low-load stretch to the spastic muscle groups, which helps prevent and reduce muscle contractures (40). Furthermore, it is also emphasizes the need for enhanced multidisciplinary collaboration and referral mechanisms (19). Regarding limited joint mobility, Evidence Item 9 integrates progressive strategies from passive release to active functional restoration, and biomechanical correction (22). Concurrently, Evidence Item 10 provides a comprehensive edema management protocol for the hand, combining exercise, positioning, and manual techniques to promote lymphatic and venous return through multiple mechanisms (22).

Evidence Items 11–12 jointly emphasize the core principle of protecting the affected limb during patient transfers (22, 41). They mandate that healthcare professionals and caregivers prioritize the biomechanical safety of the affected upper extremity during assisted mobility, preventing complications such as glenohumeral subluxation and soft tissue strain, thereby establishing a safe foundation for recovery.

### 4.4 The daily care for shoulder-hand syndrome after a stroke should be based on the principle of protection

Posture Management is essential for SHS rehabilitation. Evidence items 13–14 indicate that in patients with arm weakness, raising the arm above 90 degrees will significantly increase the risk of impact between the humeral head and the structures below the acromion, thereby causing compression and pain of the soft tissues. During wheelchair sitting and bed rest, it is necessary to ensure that the limb is not hanging, and continuous and adequate support can be provided through auxiliary devices such as pillows and arm supports to maintain joint alignment and reduce mechanical stress (22, 41). Evidence Item 15 specifically identifies devices to avoid using head-mounted arm supports, shoulder support belts and pulley devices (20). These devices may aggravate the edema and venous return obstruction to some extent, lead to the shoulder joint mechanical imbalance, and inhibit the recovery of the active movement function of the patients (42).

Evidence Items 16–18 highlight the importance of patient and caregiver education. By disseminating preventive knowledge, providing practical guidance on limb protection, and offering training in specific techniques, professional care can be effectively extended into the home setting. This approach enhances self-management capabilities and is crucial for ensuring continuity of care between hospital and community, ultimately helping to prevent complications (19, 20, 22).

### 4.5 Comparison of evidence summary with major guidelines

The best evidence integrated in this study is consistent with the practical recommendations of mainstream guidelines in core principles such as early prevention, multi-modal assessment, and comprehensive intervention strategies. However, our comprehensive analysis provides relatively detailed explanations for the specific intervention parameters of physical therapy techniques such as electroacupuncture and mirror therapy. Additionally, for certain intervention measures (such as transcutaneous electrical nerve stimulation and extracorporeal shock wave therapy), due to insufficient evidence, we maintain the same cautious recommendation attitude as the relevant guidelines, and this is also the direction for future research.

### 4.6 Limitations

This study has several limitations. First, the pathological process of post-stroke shoulder-hand syndrome is complex and dynamically evolving; however, most current evidence focuses on short-term intervention outcomes, with a scarcity of high-quality studies investigating how intervention strategies should be adapted throughout the longitudinal progression of the condition. Second, although this review has synthesized a range of effective physical therapy modalities, there remains a lack of clear, actionable, evidence-based guidance on how to select the optimal intervention based on the distinct characteristics of different disease stages. Future research should focus on elucidating the natural history of the disorder to establish staged and refined treatment pathways, thereby enhancing the precision of clinical decision-making. Furthermore, for some emerging physical therapy methods in recent years, such as extracorporeal shock wave therapy, no relevant evidence has been found to prove their effectiveness. We look forward to more scholars conducting in-depth research in these fields in the future.

## 5 Conclusion

This study systematically developed a comprehensive physical therapy management protocol for post-stroke SHS. Based on18 evidence-based items, a care framework was established centered on prevention-first principles, assessment-guided decision-making, and multimodal intervention. Early joint protection strategies can interrupt the causal chain of SHS development; standardized assessment tools provide the basis for precise interventions; symptom management adopts stage-specific combination protocols covering pain control, functional restoration, and edema reduction; while daily care and health education ensure the continuity of rehabilitation strategies. This study provides a standardized clinical care pathway spanning from hospital to home settings, with important implications for improving patient outcomes. Future studies should focus on determining optimal intervention parameters for patients at different disease stages and validating the application effectiveness of the evidence presented herein.

## Data Availability

The original contributions presented in the study are included in the article/supplementary material, further inquiries can be directed to the corresponding author/s.
