# Supplementary material for: Summary of the best evidence for physical therapy in patients with post-stroke shoulder hand syndrome
Source: Front Neurol. 2026 Apr 2;17:1779579. doi: 10.3389/fneur.2026.1779579 (PMC13082933; doi:10.3389/fneur.2026.1779579)
Supplement: Supplementary file 1 [file Table_1.docx]

**Supplementary Table 1. The literature search strategy**

| **Database** | **Search Strategy** |
| --- | --- |
| **Web of Science** | **(TS=("Physical Therapy Modalities") OR AB=(Physical Therapy Modalities OR Physical Therapy Modality OR Neurophysiotherapy OR Physical Therapy OR Rehabilitation OR training OR non-drug treatment OR prevention OR prevention OR intervention OR exercise)) AND (TS=("Stroke" OR "Brain Infarction" OR "Cerebrovascular Disorders") OR AB=(Stroke OR Brain Infarction OR Cerebrovascular Disorders OR Cerebral Stroke OR Cerebrovascular Accident OR Cerebrovascular Apoplexy OR Brain Vascular Accident OR Cerebrovascular Stroke OR Apoplexy)) AND (TS=("Reflex Sympathetic Dystrophy" OR "Complex Regional Pain Syndromes") OR AB=(Reflex Sympathetic Dystrophy OR Complex Regional Pain Syndromes OR Reflex Sympathetic Dystrophies OR Reflex Sympathetic Dystrophy Syndrome OR Cervical Sympathetic Dystrophy OR Algodystrophic Syndrome OR Shoulder Hand Syndrome))** |
| **Pubmed** | **(((((((((((((Physical Therapy Modalities[Title/Abstract]) OR (Physical Therapy Modality[Title/Abstract])) OR (Neurophysiotherapy[Title/Abstract])) OR (Physical Therapy[Title/Abstract]) ) OR (Rehabilitation[Title/Abstract])) OR (training[Title/Abstract])) OR (non-drug treatment[Title/Abstract])) OR (prevention[Title/Abstract])) OR (prevention[Title/Abstract])) OR (intervention[Title/Abstract])) OR (exercise[Title/Abstract])) OR ("Physical Therapy Modalities"[Mesh])) AND ((((((((((Stroke[Title/Abstract]) OR (Brain Infarction[Title/Abstract])) OR (Cerebrovascular Disorders[Title/Abstract])) OR (Cerebral Stroke[Title/Abstract])) OR (Cerebrovascular Accident[Title/Abstract])) OR (Cerebrovascular Apoplexy[Title/Abstract])) OR (Brain Vascular Accident[Title/Abstract])) OR (Cerebrovascular Stroke[Title/Abstract])) OR (Apoplexy[Title/Abstract])) OR ((("Stroke"[Mesh]) OR "Brain Infarction"[Mesh]) OR "Cerebrovascular Disorders"[Mesh]))) AND ((("Reflex Sympathetic Dystrophy"[Mesh]) OR "Complex Regional Pain Syndromes"[Mesh]) OR (((((((Reflex Sympathetic Dystrophy[Title/Abstract]) OR (Complex Regional Pain Syndromes[Title/Abstract])) OR (Reflex Sympathetic Dystrophies[Title/Abstract])) OR (Reflex Sympathetic Dystrophy Syndrome[Title/Abstract])) OR (Cervical Sympathetic Dystrophy[Title/Abstract])) OR (Algodystrophic Syndrome[Title/Abstract])) OR (Shoulder Hand Syndrome[Title/Abstract])))** |
| **Embase** | **'reflex sympathetic dystrophy':ab,ti OR 'complex regional pain syndromes':ab,ti OR 'reflex sympathetic dystrophies':ab,ti OR 'reflex sympathetic dystrophy syndrome':ab,ti OR 'cervical sympathetic dystrophy':ab,ti OR 'algodystrophic syndrome':ab,ti OR 'shoulder hand syndrome':ab,ti**  **AND**  **stroke:ab,ti OR 'brain infarction':ab,ti OR 'cerebrovascular disorders':ab,ti OR 'cerebral stroke':ab,ti OR 'cerebrovascular accident':ab,ti OR 'cerebrovascular apoplexy':ab,ti OR 'brain vascular accident':ab,ti OR 'cerebrovascular stroke':ab,ti OR apoplexy:ab,ti**  **AND**  **'physical therapy modalities':ab,ti OR 'physical therapy modality':ab,ti OR neurophysiotherapy:ab,ti OR 'physical therapy':ab,ti OR rehabilitation:ab,ti OR training:ab,ti OR 'non-drug treatment':ab,ti OR prevention:ab,ti OR intervention:ab,ti OR exercise:ab,ti** |
| **Cochrane Library** | **(Physical Therapy Modalities OR Physical Therapy Modality OR Neurophysiotherapy OR Physical Therapy OR Rehabilitation OR training OR non-drug treatment OR prevention OR prevention OR intervention OR exercise):ti,ab,kw**  **AND**  **(Stroke OR Brain Infarction OR Cerebrovascular Disorders OR Cerebral Stroke OR Cerebrovascular Accident OR Cerebrovascular Apoplexy OR Brain Vascular Accident OR Cerebrovascular Stroke OR Apoplexy):ti,ab,kw**  **AND**  **(Reflex Sympathetic Dystrophy OR Complex Regional Pain Syndromes OR Reflex Sympathetic Dystrophies OR Reflex Sympathetic Dystrophy Syndrome OR Cervical Sympathetic Dystrophy OR Algodystrophic Syndrome OR Shoulder Hand Syndrome):ti,ab,kw** |
| **CINAHL** | **SU (Physical Therapy Modalities OR Physical Therapy Modality OR Neurophysiotherapy OR Physical Therapy OR Rehabilitation OR training OR non-drug treatment OR prevention OR prevention OR intervention OR exercise) AND SU (Stroke OR Brain Infarction OR Cerebrovascular Disorders OR Cerebral Stroke OR Cerebrovascular Accident OR Cerebrovascular Apoplexy OR Brain Vascular Accident OR Cerebrovascular Stroke OR Apoplexy) AND SU (Reflex Sympathetic Dystrophy OR Complex Regional Pain Syndromes OR Reflex Sympathetic Dystrophies OR Reflex Sympathetic Dystrophy Syndrome OR Cervical Sympathetic Dystrophy OR Algodystrophic Syndrome OR Shoulder Hand Syndrome)** |
| **Scopus** | **All fields (Physical Therapy Modalities OR Physical Therapy Modality OR Neurophysiotherapy OR Physical Therapy OR Rehabilitation OR training OR non-drug treatment OR prevention OR prevention OR intervention OR exercise) AND**  **All fields (Stroke OR Brain Infarction OR Cerebrovascular Disorders OR Cerebral Stroke OR Cerebrovascular Accident OR Cerebrovascular Apoplexy OR Brain Vascular Accident OR Cerebrovascular Stroke OR Apoplexy)**  **AND**  **All fields (Reflex Sympathetic Dystrophy OR Complex Regional Pain Syndromes OR Reflex Sympathetic Dystrophies OR Reflex Sympathetic Dystrophy Syndrome OR Cervical Sympathetic Dystrophy OR Algodystrophic Syndrome OR Shoulder Hand Syndrome)** |
