# Supplementary material for: Summary of the best evidence for physical therapy in patients with post-stroke shoulder hand syndrome
Source: Front Neurol. 2026 Apr 2;17:1779579. doi: 10.3389/fneur.2026.1779579 (PMC13082933; doi:10.3389/fneur.2026.1779579)
Supplement: Supplementary file 2 [file Table_2.docx]

**Supplementary Table 2 The quality evaluation of Clinical decision and Evidence Synthesis**

| **Items** | **[Fan et al.](https://pmc.ncbi.nlm.nih.gov/articles/PMC12312446/#CR2)**  **[(23)](https://pmc.ncbi.nlm.nih.gov/articles/PMC12312446/#CR2)** | **Saikaley et al.**  **(24)** |
| --- | --- | --- |
| 1. Is the summary specific in scope and application? | Yes | Yes |
| 2. Is the authorship of the summary transparent? | Yes | Yes |
| 3. Are the reviewer(s)/editor(s) of the summary transparent? | Yes | Yes |
| 4. Are the search methods transparent and comprehensive? | No | Yes |
| 5.Is the evidence graded and is the system transparent and translatable? | Yes | Yes |
| 6. Are the recommendations clear? | Yes | Yes |
| 7. Are the recommendations appropriately cited? | Yes | Yes |
| 8. Are the recommendations current? | Yes | Yes |
| 9. Is the summary free of possible bias? | Yes | Yes |
| 10. Can this summary be applied to your patient(s)? | Yes | Yes |
