# Supplementary material for: Summary of the best evidence for physical therapy in patients with post-stroke shoulder hand syndrome
Source: Front Neurol. 2026 Apr 2;17:1779579. doi: 10.3389/fneur.2026.1779579 (PMC13082933; doi:10.3389/fneur.2026.1779579)
Supplement: Supplementary file 3 [file Table_3.docx]

**Supplementary Table 3 The quality evaluation of systematic reviews**

| Items | Liu (8) | Peng (25) | Lei (26) | Shi (27) | Huang (28) | Wei (29) | Wang  (30) | Yu (31) | Shafiee (32) | Kanika(33) | Feng  (34) | Gao (35) | Meng (36) | Wang  (37) |  |
| --- | --- | --- | --- | --- | --- | --- | --- | --- | --- | --- | --- | --- | --- | --- | --- |
| *1.Did the research questions and inclusion criteria for the review include the components of PICO?* | Yes | Yes | Yes | Yes | Yes | Yes | Yes | Yes | Yes | Yes | Yes | Yes | Yes | Yes |  |
| *2.Did the report of the review contain an explicit statement that the review methods were established prior to the conduct of the review and did the report justify any significant* *deviations from the protocol?* | Yes | Yes | Yes | Yes | Yes | No | No | No | Yes | Yes | Yes | Yes | Yes | Yes |  |
| *3.Did the review authors explain their selection of the study designs for inclusion in the review?* | Yes | Yes | Yes | Yes | Yes | Yes | Yes | Yes | Yes | Yes | Yes | Yes | Yes | Yes |  |
| *4.Did the review authors use a comprehensive literature search strategy?* | Yes | Yes | Yes | Yes | Yes | Yes | Yes | Yes | Yes | Yes | Yes | Yes | Yes | Yes |  |
| *5.Did the review authors perform study selection in duplicate?* | Yes | Yes | Yes | Yes | Yes | Yes | Yes | Yes | Yes | Yes | Yes | Yes | Yes | Yes |  |
| *6.Did the review authors perform data extraction in duplicate?* | Yes | Yes | Yes | Yes | Yes | Yes | Yes | Yes | Yes | Yes | Yes | Yes | Yes | Yes |  |
| *7.Did the review authors provide a list of excluded studies and justify the exclusions?* | Yes | Yes | Yes | Yes | Yes | Yes | Yes | Yes | Yes | Yes | Yes | Yes | Yes | Yes |  |
| *8.Did the review authors describe the included studies in adequate detail?* | Yes | Yes | Yes | Yes | Yes | Yes | Yes | Yes | Yes | Yes | Yes | Yes | Yes | Yes |  |
| *9.Did the review authors use a satisfactory technique for assessing the risk of bias (RoB) in individual studies that were included in the review?* | Yes | Yes | Yes | Yes | Yes | Yes | Yes | Yes | Yes | Yes | Yes | Yes | Yes | Yes |  |
| *10.Did the review authors report on the sources of funding for the studies included in the review?* | No | No | No | No | No | No | No | No | No | No | No | No | No | No | No |
| *11.If meta-analysis was performed, did the review authors use appropriate methods for statistical combination of results?* | Yes | Yes | Yes | Yes | Yes | Yes | Yes | Yes | Yes | Yes | Yes | Yes | Yes | Yes |  |
| *12.If meta-analysis was performed, did the review authors assess the potential impact of RoB in individual studies on the results of the meta-analysis or other evidence synthesis?* | Yes | Yes | Yes | Yes | Yes | Yes | Yes | Yes | Yes | Yes | Yes | Yes | Yes | Yes |  |
| *13.Did the review authors account for RoB in primary studies when interpreting/discussing the results of the review?* | Yes | Yes | Yes | Yes | Yes | Yes | Yes | Yes | Yes | Yes | Yes | Yes | Yes | Yes |  |
| *14.Did the review authors provide a satisfactory explanation for, and discussion of, any heterogeneity observed in the results of the review?* | Yes | Yes | No | Yes | Yes | Yes | Yes | Yes | Yes | Yes | Yes | Yes | Yes | Yes |  |
| *15.If they performed quantitative synthesis did the review authors carry out an adequate investigation of publication bias (small study bias) and discuss its likely impact on the results of the review?* | Yes | Yes | Yes | Yes | Yes | Yes | Yes | Yes | No | No | Yes | Yes | Yes | Yes |  |
| *16.Did the review authors report any potential sources of conflict of interest, including any funding they received for conducting the review?* | Yes | Yes | Yes | Yes | Yes | Yes | Yes | No | Yes | Yes | Yes | Yes | Yes | Yes |  |
